# Passive UHF RFID Tag with Multiple Sensing Capabilities

**DOI:** 10.3390/s151026769

**Published:** 2015-10-22

**Authors:** José Fernández-Salmerón, Almudena Rivadeneyra, Fernando Martínez-Martí, Luis Fermín Capitán-Vallvey, Alberto J. Palma, Miguel A. Carvajal

**Affiliations:** 1 The Institute for Nanoelectronics, Technical University of Munich, DE-80333 Munich, Germany; E-Mails: jfsalmeron@nano.ei.tum.de (J.F.-S.); arivadeneyratorres@nano.ei.tum.de (A.R.); 2 Department of Electronic and Computer Technology, ECSens, University of Granada, E-18071 Granada, Spain; E-Mails: fmartinezmarti@ugr.es (F.M-M.); ajpalma@ugr.es (A.J.P.); 3 Analytical Chemical Department, ECSens, University of Granada, E-18071 Granada, Spain; E-Mail: lcapitan@ugr.es

**Keywords:** screen printing, printed electronics, UHF antenna, RFID tag, sensor, switch

## Abstract

This work presents the design, fabrication, and characterization of a printed radio frequency identification tag in the ultra-high frequency band with multiple sensing capabilities. This passive tag is directly screen printed on a cardboard box with the aim of monitoring the packaging conditions during the different stages of the supply chain. This tag includes a commercial force sensor and a printed opening detector. Hence, the force applied to the package can be measured as well as the opening of the box can be detected. The architecture presented is a passive single-chip RFID tag. An electronic switch has been implemented to be able to measure both sensor magnitudes in the same access without including a microcontroller or battery. Moreover, the chip used here integrates a temperature sensor and, therefore, this tag provides three different parameters in every reading.

## 1. Introduction

One emerging technology to control the supply chain is radio frequency identification (RFID). The vast majority of RFID tags consist of an antenna and an integrated circuit (IC). Most RFID tags are low-cost passive tags, which take advantage of the reader-induced signal to supply power to the RFID chip [1]. In this sense, printed electronics results in a good alternative to produce low-cost systems in large-scale production. Several examples are found of RFID tags developed with different printing techniques such as screen printing, gravure, flexography, or inkjet printing [2,3,4,5]. In addition to cost savings, printed electronics can produce thin, wearable, and lightweight devices, as well as flexible systems by using malleable substrates [6].

In recent years, there has been a growing interest in developing RFID tags with sensing capabilities. Different approaches have been followed to achieve this goal. On one side, some authors have studied the analog response of the sensing RFID tag: read range, shift of the antenna resonance frequency, measured level of backscattered power, *etc*. Changes in these electrical parameters have been associated to a variation of the monitored magnitude [2,7,8,9,10,11,12,13,14,15,16,17,18], resulting in useful strategies as threshold sensor tags [19] but they lead to uncertainties introduced by the wireless link between RFID reader and tag. It is difficult to know whether a change in the analog response of the tag can be directly related to a variation in the monitored magnitude or it is affected by other factors that interfere in the link performance, such as path loss between antennas, spatial and time drifts, electrical interference, non-direct sight between reader and tag, interferences with other objects, *etc*. Furthermore, extra-circuitry typically has to be included into the RFID reader to monitor the tag analog response. On the other hand, other approaches are based on microcontroller architectures in combination with RFID chips and different types of sensors: temperature, light, and moisture content [20,21]; chemical sensing [22,23,24,25]; printed moisture sensors [26,27]; pressure [28,29]; general RFID platforms for different sensing applications [30,31], SAW sensors [32] or built-in sensors, commonly temperature sensors [33]. Additionally, some examples of single-chip architecture without microcontroller units have been reported by [34,35,36,37]. The main advantage of this strategy compared to the analog approach is the processing of the sensor data directly in the RFID tag. Thus, the sensor data, in digital form, can be reliably delivered to the reader or stored on the tag for future access. Nevertheless, these strategies require the use of a battery to enable data logging and power the necessary extra-chips, such as a microcontroller unit to drive the different sensors. All these extra components increase the cost of the system. 

Here, we present the design, fabrication and characterization of a printed RFID tag with sensing capabilities. This passive tag is printed on a cardboard box with the aim of checking the packaging conditions. The tag includes a commercial force sensor and a printed opening detector. In this sense, the mass applied to the packaging can be measured as well as the detection of the opening of the box. These two sensors provide relevant information during all stages of the supply chain. For example, shock events and applied forces invariably occur in the shipping industry but they are difficult to be detected by different stakeholders. For instance, a study showed that a package would experience, on average, a drop from a height of 1–1.85 m during its transit [38] and another study found that an average commercial truck would induce loads from 5–35 g [39,40]. Sensors could be mounted on shipping containers or packages to determine if any force has been applied in its transit through the supply chain [41].

As remarkable features, the architecture presented here is based on a single-chip architecture using screen printing technology as fabrication process. In order to use a unique chip with two sensors, an electronic switch has been included in the tag that reads them independently. Thus, both sensor magnitudes can be measured in the same access without including a microcontroller or battery. Our tag only requires several external transistors and resistors to obtain sensor data. Due to the passive architecture, it is necessary to power up the tag from the electromagnetic field radiated by the RFID reader. Obviously, the opening detector has memory (once it is broken, it is not possible to recover it), whilst the force pressure sensor provides information about the mass applied at the moment of the sensor reading. Moreover, due to the fact that the chip used here integrates a temperature sensor, these tags provide three parameters in every reading. Regarding the frequency band, the ultra-high frequency band (UHF) has been chosen here for its higher read range and the possibility of simultaneous detection of several tags. The high frequency (HF) band could have interesting results with the implementation of the near-field communication (NFC) protocol that many mobile phones already include, and therefore no specific reader would be required. The drawback of this band is its shorter read range, no more than few centimeters.

## 2. Material and Method

### 2.1. Smart Tag Architecture

Two resistive sensors are integrated in this tag: a force sensor and an opening detector. In order to measure the vertical force applied to the packaging, we selected the FlexiForce A201 force sensor (Tekscan, Inc., South Boston, MA, USA) working in the force range up to 111 N (25 pounds). This sensor presents an error lower than 3% and its hysteresis is lower than 4.5%. Its time response is below 5 µs. As an opening detector, we printed a conductive pattern through the die cut line of the package. In this sense, we are able to measure a low resistance value when the package is closed, whereas when it is opened, this printed pattern is broken and, therefore, an open circuit is detected. 

Temperature value comes from a conversion in the on-chip A/D converter of the SL900A. Two internal voltage references, V_ref1_ and V_ref2_ individually selectable in steps of 50 mV between 160 and 610 mV, set the lower and upper limits of this converter. These limits are defined as 2V_ref1_–V_ref2_ and V_ref1_. The difference between them defines the input voltage range, V_ref2_–V_ref1_, and the limits of operation. These voltage references can be set in the user application and, therefore, a concrete resolution and range can be selected by the user. The minimum resolution of the built-in temperature sensor is 0.18 °C in a range from −89.3 °C to 84.6 °C, while a resolution of 0.23 °C is obtained with the widest range from −89.3 °C to 147.9 °C. It should be noted that operation of the RFID chip below −40 °C and above 125 °C is not guaranteed by the manufacturer.

**Figure 1:**
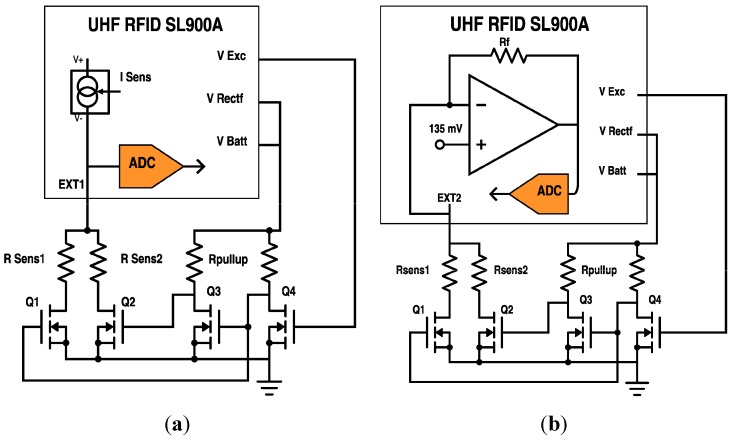
Schematics showing the two external resistive sensor modes of the RFID chip, including the extra circuitry to read each resistive sensor: (**a**) sensor with linear resistance variation; and (**b**) sensor with linear conductance variation.

The resistive sensors are directly connected to the sensor front end (SFE) of the RFID chip. To read out a resistance value, it does not require any external components but, in our case, we want to measure sequentially two different resistive sensors in the same access. For this purpose, a switch based on discrete MOS transistors has been implemented (see Figure 1). The transistors are BSS138LT1 NMOS (Semiconductor Components Industries LLC, USA) with a resistance value in on-region R_DS(on)_ = 3.5 Ω and SMD pull-up resistors of 4.7 kΩ (Panasonic, Japan). V_EXC_ is the switch control signal that is controlled by the RFID reader. V_EXC_ terminal, which is usually employed as voltage supply for external sensors, can be set to high or low level. In this application, V_EXC_ is used as the switch control signal: when V_EXC_ is set at the low level, the sensor R_Sens1_ is selected, whereas R_Sens2_ is measured when high level is set in V_EXC_ (Figure 1). Therefore, only one sensor is read each time. Two inverters (Q3 and Q4) are needed in order to ensure the maximum available voltage in the gates of Q1 and Q2 and reduce the ON resistance, R_DS(on)_, of these transistors. In fact, the measured R_DS(on)_ value is only a few Ω that is negligible in comparison with the sensors resistance, as will be shown below.

**Figure 2:**
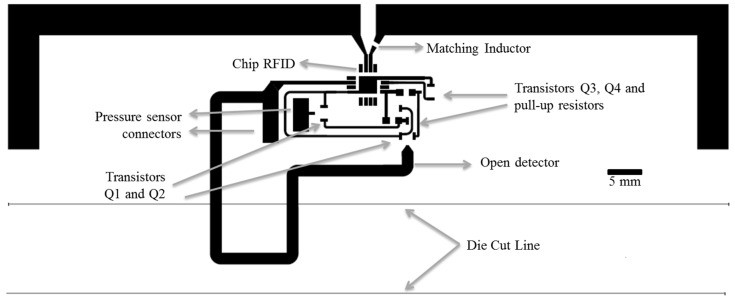
Layout of the RFID tag comprising a dipole antenna, an RFID chip and the sensor architecture. Components have been labeled for better understanding.

Figure 2 shows the layout of the printed tag, including the footprints of the different components required. This tag presents a passive architecture based on SL900A RFID chip (AMS AG, Unterpremstaetten, Austria) compatible with EPC Gen 2 RFID standard [42]. The RFID chip was chosen due to its integration of a SFE that comprises different sensor conditioning stages and a 10-bit analog-to-digital converter (ADC). Extra circuitry has been used to interface the resistive sensors. In addition, this RFID chip includes an in-built temperature sensor. The radio frequency interface consists of a typical dipole antenna resonating at 868 MHz (European UHF RFID band) and a RF surface mount device (SMD) inductor used to match the chip input impedance [43]. The dipole antennas are the best candidate for RFID applications because of its nearly omnidirectional character, *i.e*., labels can be detected in almost any relative position of the RFID reader to the antenna. In case of choosing a different frequency band, it would be only necessary to change the antenna layout and the RFID chip.

In order to read out the smart tag by a RFID reader, several EPC Gen 2 custom commands must be sent to select V_EXC_ level and different parameters to read each resistive sensor. In this particular case, *Set_SFE_Parameters* command is employed to choose the SFE configuration and *Set_Calibration_Data* command is sent to configure the ADC reference and V_EXC_ signal. Another EPC Gen 2 command, *Get_Sensor_Value*, is used then to get the resistance value. EPC Gen 2 command delay must be lower than 20 ms according to protocol specifications. This delay depends on the selected data rate (5–640 kbits/s); forward and backward links operation between RFID reader and tag and the number of tags detected by the reader. An extra time of 6 ms is employed by the ADC converter to acquire and process the resistance values in the case of *Get_Sensor_Value* command. Due to the fact that this process is realized sequentially to read out each resistive sensor, the total time required, assuming the worst case (maximum delay between reader and tag), is below 66 ms.

Two different SFE configurations are used to read out the resistive sensors: linear resistance and linear conductance. The former configuration (Figure 2a) is indicated for sensors whose resistance varies linearly with the sensed magnitude. The sensor resistance selected by the switch is polarized with a configurable current source (I_SENS_) whose amplitude can be selected from 0 to 7.75 µA, in steps of 250 nA. The input voltage of the ADC, V_ADC_, is associated with the resistance value as follows:

(1) $$V_{ADC}=I_{SENS}\left(R_{SENS,i}+R_{DS\left(on\right)}\right)$$

In order to measure both sensors, it is necessary to properly select the current value; the opening detector was measured with a current equal to I_SENS_ = 7 µA, whilst the force sensor was configured with I_SENS_ = 6 µA. This difference in current is given by the range of resistance variation; in the opening detector case, it is enough to detect a change from a few Ω (close state) to open circuit (open state). In the force sensor case, the resistance value, which varies from some MΩ (no pressure) to a few kΩ, is measured to determine the applied force. As previously explained, the limits of the ADC input voltage are V_ref1_ and 2V_ref1_–V_ref2_. In the case of the opening detector, these ADC references are set to V_ref1_ = 260 mV and V_ref2_ = 0 V, so the ADC input voltage ranges from 260 mV to 520 mV leading to a detection range of resistance from 37 to 74 kΩ. For the force pressure sensor, the ADC references are set to V_ref1_ = 610 mV and V_ref2_ = 0 V, giving an ADC input voltage range of 610–1220 mV that corresponds to a detection range of 100–203 kΩ for a sensing current of 6 µA. Any other detection range could be easily configured by choosing the appropriate sensing current and ADC limits.

The latter readout configuration (Figure 2b) is suitable for sensors whose conductance changes linearly with the sensed magnitude. This is the case of the force sensor used in this work. An operational amplifier with negative feedback is combined with a selectable feedback resistor R_FB_ (185, 400, 875, 1875 or 3875 kΩ), see Figure 2b. The non-inverting input is fixed to 135 mV, so the input voltage in the ADC is related to R_SENS_ value:

(2) $$V_{ADC}=135\;mV+135\;mV\;\frac{R_{FB}}{R_{SENS,i}}$$

For optimal operation, the feedback resistor value and the ADC reference voltages have been chosen as close as possible to the resistive sensor values and the non-inverting input voltage, respectively: the feedback resistor is 185 kΩ whilst V_ref1_ is 260 mV and V_ref2_ is set to 0 V. This configuration leads to a detection range for the resistive sensors from 65 to 200 kΩ.

### 2.2. Fabrication Process

All the patterns were directly printed on a commercial cardboard box (Sociedad Estatal Correos y Telégrafos, S.A., Madrid, Spain) with dimensions 317 × 215 × 125 mm and thickness of 1.5 mm. Screen printed patterns were manufactured with a Serfix III screen printing machine (Seglevint SL, Barcelona, Spain). The screen used to manufacture the tag had a mesh count of 120 Nylon thread per centimeter (T/cm). The prototype was fabricated with only one screen printed layer of conductive silver ink CRSN 2442 (Sun Chemical Corporation, Parsippany, NJ, USA). The minimum spatial resolution for a reliable fabrication process was 150 μm. Sintering took place at a constant temperature of 80 °C for 20 min [43].

Finally, the assembly of the chips and external components to the cardboard box was carried out with a three-step process. First, the interconnections between RFID chip and silver pads were made by using the conductive resin H20E (Epoxy Technology, Inc., Billerica, MA, USA). A double layer of 50 μm-thick dry adhesive, AR Clear 8932 (Adhesives Research, Inc., Glen Rock, NJ, USA), was placed on the bottom of the chip to fix it to the substrate. Finally, the conductive resin was cured by heating in an oven at 120 °C for 20 min. Moreover, the dry film adhesion improved with temperature, so this heat treatment served also to better fix the components to the substrate.

## 3. Results and Discussion

### 3.1. Antenna Characterization

**Figure 3:**
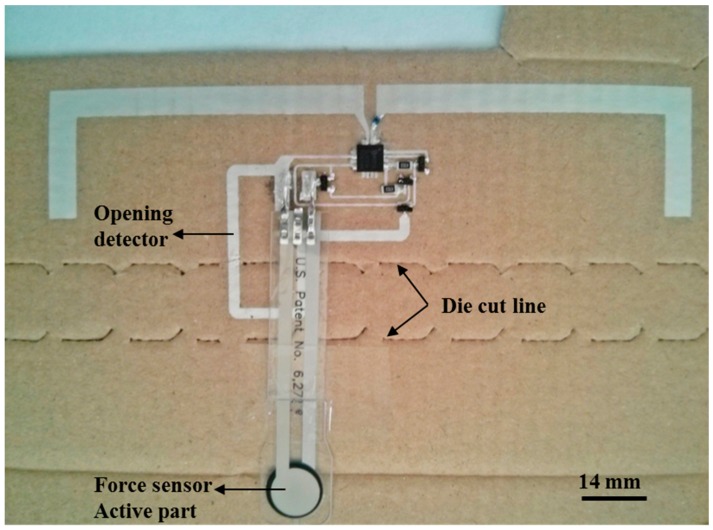
Fabricated RFID tag with all components.

Figure 3 presents the manufactured RFID tag with its different components labeled for better understanding. Final dimensions of the screen printed dipole antenna arms are 5.5 mm width and 72 mm length. The thickness and roughness of the printed layers were measured with the Dektak XT™ Stimulus Surface Profiling System (Bruker Corporation, Conventry, UK), finding a thickness of 17 ± 2 µm with a root mean square (RMS) roughness of 1.7 µm. In order to optimize the occupied area, the dipole arms have been bent. The input impedance of the dipole is (31.1 + j9.4) Ω at 868 MHz. The impedance of this dipole has been designed to achieve the same real part as the one of the RFID chip, whilst the necessary large inductive part is achieved with a SMD inductor series 3650 (TE Connectivity, Ltd., Schaffhausen, Switzerland) placed on one of its arms. Its inductance is 51 nH and its quality factor is 60 at 900 MHz. The antenna parameters have been obtained by EM simulation with Advanced Design System 2013 (Keysight Technologies Inc., Santa Rosa, CA, USA). Substrate influence has been included in the simulations and its electrical parameters have been taken from [31]. In particular, its gain is 0.661 dBi, its directivity 2.401 dBi and its efficiency 66.97% at the working frequency [29].

According to the theory of communication in RFID systems [43,44], the maximum read range is calculated using Equation (3).

(3) $$range_{max}=\frac{\lambda }{4\pi }\sqrt{\frac{G_{tag}G_{reader}P_{reader}\tau \;PLF}{S_{tag}}\;}$$

where S_tag_ is the RFID chip sensitivity; the minimum received power level to activate the tag is −15 dBm for the chosen RFID chip. G_tag_ refers to the tag antenna gain, P_reader_ is the effective radiated power by the reader and G_reader_ the reader antenna gain. The polarization mismatch between the reader antenna (circular polarization) and the designed tag antenna (lineal polarization) is also included. This is represented as the path loss factor (PLF) in Equation (3) and its value is 0.5 (3 dB). The transmission power is 26 dBm and the reader antenna gain is 7 dBi at 868 MHz, according to the manufacturer. The factor τ takes into account the losses related to the mismatch between the chip and the antenna [43]. 

The measurements of the maximum read range took placed on anechoic chamber; the antenna reader was placed on a tripod whilst the RFID tags printed on the cardboard box were placed at the same height. RFID tag tests were performed using a commercial RFID reader compatible with EPC Gen 2, DK-UHF RFID HP2 (IDS Microchip AG, Wollerau, Switzerland). The read range is the maximum distance in which the reader is able to read out the tag including the sensing functionalities. It should be noted that the sensitivity of the RFID chip has a penalty. The chip sensitivity to answer identification inquiries of EPC protocol is −15 dBm, whereas the sensitivity to read out a value of resistance is −2.21 dBm. The reason to explain these differences is the fact that extra power is required to drive the SFE. This extra power is collected from the radiated EM field of the reader. This aspect has been already studied in our previous works [29,37] and it is calculated using the antenna parameters and on-chip RF rectifier efficiency, ~40% according to the manufacturer.

According to Equation (3) and setting chip sensitivity to −2.21 dBm, the maximum read range should be 2.2 m, assuming ideal conditions (perfect matching between chip, τ = 1, and the dipole antenna gain obtained by EM simulation, G_tag_ = 0.66 dBi). However, the measured range for the printed tag at room conditions is smaller than the simulated one, 1.1 m. These differences between simulated and measured read ranges were already reported on our previous work [29], being attributed to the following factors: the non-ideal behavior of the metallic layers in printed electronic that leads to a lower gain and efficiency of the fabricated antennas [29] and extra losses introduced by the mismatch between the impedances of RFID chip and antenna because of the variation of the RFID chip impedance with both working frequency and powers levels. In this case, the read range has been reduced approximately two thirds compared with the same antenna fabricated on polyimide substrate [29]. Although the thick cardboard substrate influence has been taken in account, this reduction could be associated with extra losses and detuning of the RFID antenna operating on paper substrate [31]. Moreover, there is a non-direct sight between RFID reader and tag antenna because the whole tag is printed on the inner side of the cardboard box. To justify this reduction, a commercial UHF RFID Raflatac tag from UPM RFID (SMARTRAC N.V., Amsterdam, The Netherlands) has been detected on the same conditions: a direct line of sight to the RFID reader on free air condition and attached to the inner side of the cardboard box. In these scenarios, the measured read ranges were 3.3 m and 2.28 m, respectively. In this case, the read ranges are larger due to the lower threshold voltage to activate these RFID tags, but the read range is penalized in the same way as our dipole antenna explaining the results found.

### 3.2. Sensing Tag Performance

**Figure 4:**
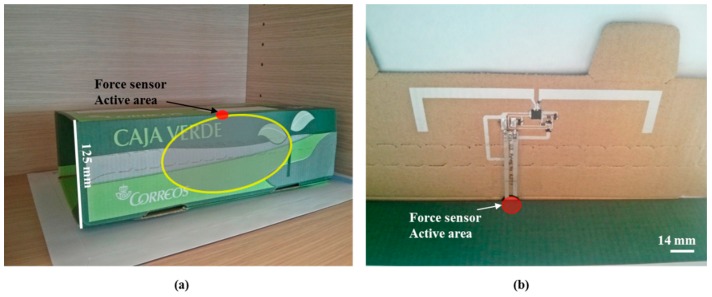
(**a**) Close cardboard with RFID tag; (**b**) view of the RFID tag before closing the box.

Figure 4 shows the cardboard box with the printed RFID tag. Regarding the force sensor, its active part has been placed just in the junction part of the cover and lateral of the box (Figure 4b). When some weight is applied to the top of the box, the pressure force is distributed among the junction between the cover and lateral sides of the box. Anyway, the force sensor can be placed where the manufacturer considers more critical. With respect to the opening detector, it has been defined through the die cut line in such a way that once the box is closed, to open it again, it is necessary to strip the die cut line off. Therefore, the opening detector is obviously non-reversible.

Temperature measurements are directly obtained from the on-chip sensor and present different errors and accuracy depending on the selected measurement range as mentioned above. 

After setting the different configurations, we have characterized the opening detector and force pressure sensor using the two presented configurations: linear resistive and linear conductive modes (see Figure 2a,b, respectively), as well as the in-built temperature sensor.

Regarding the opening detector in linear resistive mode, the maximum ADC counts are read when the packaging is opened. On the contrary, the zero level is returned while the box is closed. No error was observed in any of the performed test. A similar behavior has been found in the linear conductance mode, being the maximum ADC counts achieved in the case of a closed package and zero when the package has been opened. This behavior is expected due to the fact that the two resistance values (a few Ω and open circuit) are out of ADC limits, in both cases.

**Figure 5:**
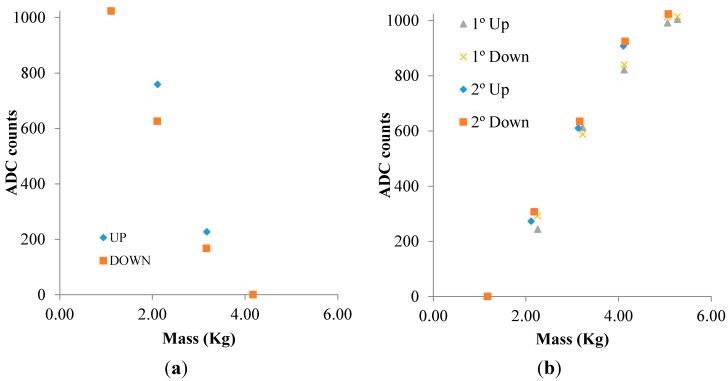
ADC counts for the force sensor measurements: (**a**) configuration as linear resistance; and (**b**) configuration as linear conductance.

Regarding the force sensor, a mass has been applied to the top of the force sensor. Mass has been measured with a weighing scale model Soehnle 67080 (Leifheit AG, Nassau, Germany) with a resolution of 1 g. We have stored and averaged fifteen ADC data to calculate the deviation between measurements. This deviation has been lower than 15 Least Significant Bit (LSB) of the ADC in all the studied cases. Figure 5 presents the average data obtained in the ADC for the force sensor on both operation modes. The output range for the force sensor is equal to the maximum resolution of the internal ADC, that is to say, 1024 counts can be associated with a variation of about 4.17 kg in the first SFE configuration (Figure 5a), whereas this output range in the second configuration (Figure 5b) corresponds to a change of about 5.27 kg. This force sensor presents a resistance of some MΩ when there is no mass on it, decreasing its value while increasing the mass. Detection limits are different due to the different detection ranges of each configuration. In the first case, we have been able to discriminate values from 1 kg to 4 kg, whilst in the second case, the lower limit is 1.2 kg and the upper limit is 5 kg. With respect to the hysteresis of the measurements, the maximum error found in both configurations is about 3%, which is within the error of the force sensor.

The conductive epoxy used in this work has a resistivity below 0.4 mΩ·cm and was deposited using a 140 T/cm screen that gets ~10 µm thickness, see [45], leading to a sheet resistance of roughly 4 Ω/□. Screen silver patterns gets a resistivity in the range of 40–50 mΩ·cm or, equivalently, a sheet resistance of 0.02–0.03 Ω/□. Although this difference is important, the epoxy has been used only in the chip pins (size of 0.5 mm width and 1.4 mm length), pressure sensor interconnection (2.6 mm width and 5.6 mm length), and switch transistors pads (1.2 mm width and 0.4 mm length). Using the calculated epoxy sheet resistance and taking into account that there are several connections for each resistive sensor; the increase in resistance is about 26 Ω for the opening detector sensor and about 42 Ω for the force sensor. In the case of the force sensor, this increment in resistance is neglected compared to its intrinsic resistance. In the case of the opening detector sensor, this increment in resistance is not critical because the sensor changes from several kΩs to open-state condition. The on-chip sensor front end and ADC are powered internally from the internal rectifier so epoxy resin only affects to antenna–RFID chip connections. This aspect has been fully studied in [29] and RFID chip input impedance was measured using the epoxy as the bonding material, so its influence has been taken into account and fully studied in [29].

Finally, the tag was introduced in the climatic chamber VCL 4006 (Vötsch Industrietechnik GmbH, Balingen, Germany) and the temperature was programmed to decrease from 30 °C to 20 °C in 10 min at RH room conditions. The temperature measurement difference between the chamber sensor and tag sensor always was below 0.5 °C, which is covered by their respective uncertainties.

## 4. Conclusions

This work describes a dipole-based UHF RFID tag with multiple sensor capabilities integrated on a cardboard package. This tag has been manufactured by screen printing without any pre-treatment of the cardboard. A simple conditioning circuit has been designed and tested to allow the multiplexing of one SFE input. This solution improves the sensing capability of this kind of RFID chip. In this work, two resistance sensors are included in the design: a silver trace crossing the die cut line of the package to detect if the package has been opened and one force sensor to measure how much weight is applied to the top of the package. In particular, the selected force sensor is able to discriminate weights in the range of 1 to 5 kg, depending on the sensing configuration mode and selected parameters. Furthermore, the chosen RFID chip includes an in-built temperature sensor. Therefore, three different magnitudes can be measured in every reading with neither microcontroller nor battery.

In order to connect both sensors to the single-sensor interface of the RFID chip without including a microcontroller, a switch has been integrated on this design. Four transistors compose this switch: two transistors to control which resistive sensor is accessible each time and two inverters to ensure the maximum available voltage in the gates of the control transistors. 

The operation of this passive tag has been successfully demonstrated. Two different modes of acquiring resistance values have been tested: linear conductance and linear resistance. The opening detector works well in both configurations. Regarding the force sensor, the linear conductance configuration results are more appropriate because a larger detection range, better linearity, and lower hysteresis are achieved compared with the linear resistance configuration. This passive tag would be very useful to detect the package conditions during the different stages of the supply chain where an RFID reader is available.
